# Bone marrow mesenchymal stem cells enhance autophagy and help protect cells under hypoxic and retinal detachment conditions

**DOI:** 10.1111/jcmm.15008

**Published:** 2020-01-30

**Authors:** Xin Liu, Jia'nan Xie, Longfei Yang, Ying Li, Yuxi He, Zaoxia Liu, Yan Zhang, Guanfang Su

**Affiliations:** ^1^ Eye Center The Second Hospital of Jilin University Changchun China; ^2^ Jilin Provincial Key Laboratory on Molecular and Chemical Genetic The Second Hospital of Jilin University Changchun China

**Keywords:** apoptosis, autophagy, bone marrow mesenchymal stem cells, hypoxia, photoreceptor cell damage, retinal detachment

## Abstract

Our study aimed to evaluate the protective role and mechanisms of bone marrow mesenchymal stem cells (BMSCs) in hypoxic photoreceptors and experimental retinal detachment. The cellular morphology, viability, apoptosis and autophagy of hypoxic 661w cells and cells cocultured with BMSCs were analysed. In retinal detachment model, BMSCs were intraocularly transplanted, and then, the retinal morphology, outer nuclear layer (ONL) thickness and rhodopsin expression were studied as well as apoptosis and autophagy of the retinal cells. The hypoxia‐induced apoptosis of 661w cells obviously increased together with autophagy levels increasing and peaking at 8 hours after hypoxia. Upon coculturing with BMSCs, hypoxic 661w cells had a better morphology and fewer apoptosis. After autophagy was inhibited, the apoptotic 661w cells under the hypoxia increased, and the cell viability was reduced, even in the presence of transplanted BMSCs. In retina‐detached eyes transplanted with BMSCs, the retinal ONL thickness was closer to that of the normal retina. After transplantation, apoptosis decreased significantly and retinal autophagy was activated in the BMSC‐treated retinas. Increased autophagy in the early stage could facilitate the survival of 661w cells under hypoxic stress. Coculturing with BMSCs protects 661w cells from hypoxic damage, possibly due to autophagy activation. In retinal detachment models, BMSC transplantation can significantly reduce photoreceptor cell death and preserve retinal structure. The capacity of BMSCs to reduce retinal cell apoptosis and to initiate autophagy shortly after transplantation may facilitate the survival of retinal cells in the low‐oxygen and nutrition‐restricted milieu after retinal detachment.

## 1 INTRODUCTION

Retinal detachment (RD), defined as the separation of neurosensory retina (including photoreceptors) from the underlying retinal pigment epithelium (RPE) which transport nutrients (including glucose) to photoreceptors, is one of the most common sight‐threatening eye diseases.^1^, ^2^, ^3^ The separation may lower the supply of oxygen and nutrients to photoreceptor out segments, rendering a relative hypoxic milieu which may further impair the energy production required for nutrients transport to photoreceptors, thus diminishing the photoreceptor function.^1^, ^3^ Hypoxia can also engender oxidative stress, causing photoreceptor apoptosis.^1^, ^4^ In addition, inflammatory cytokines released in RD also contribute to photoreceptor death.^5^ Although the anatomic reattachment rate has greatly increased with advances in surgical management, impaired visual acuity may also occur due to photoreceptor apoptosis, necrosis, autophagy, retinal remodelling together with other structural and functional retinal changes.^1^, ^5^, ^6^, ^7^ Attempts to save photoreceptors have been made, including suppressing factors participating in apoptosis, providing neurotrophic factors and modulating inflammation. However, as photoreceptor death is mediated by multiple parallel pathways, strategies aiming at a single factor are not always completely valid.^1^, ^2^, ^8^, ^9^

Bone marrow mesenchymal stem cells (BMSCs), acting mainly as the progenitors of all connective tissue cells, can differentiate into several tissue‐forming cells and play a vital role in retinal cell regeneration.^10^, ^11^, ^12^ Many studies have also demonstrated that BMSCs exert anti‐apoptotic and anti‐inflammatory effects and represent a promising strategy for the treatment of retinal diseases. Mechanisms underlying the effects of BMSCs include but were not limited to their ability to express a variety of cytokines and neurotrophic factors.^13^, ^14^ BMSCs were also reported to actively participate in autophagy to ameliorate ischaemia/reperfusion‐induced lung injury and liver fibrosis.^15^, ^16^ More important, BMSCs were reported to protect ischaemic retina by increasing autophagy.^17^ However, there has been no report on the effects of BMSCs on photoreceptor damage in retinal detachment. Thus, our study was to investigate whether the BMSCs could protect detached and hypoxic retina and whether autophagy participates in the effects of BMSCs on retina.

Our study here suggested that BMSCs play a protective role against photoreceptor death in experimental RD. We also studied the mechanisms by which these stem cells protect photoreceptors in vitro and in vivo.

## 2 MATERIALS AND METHODS

### 2.1 Cell culture

Rat BMSCs were isolated from bone marrow stroma harvested from the femurs and tibias of female Wistar rats (7 days after birth) as described.^18^ The cells were seeded in Dulbecco's modified Eagle's Medium (DMEM)‐F12 (HyClone) supplemented with 10% foetal bovine serum (FBS, Gibco) at 37°C in humified air with 5% CO_2_. Rat BMSCs were phenotypically characterized and identified as described,^19^ as Figure S1 for BMSC identification. Bone marrow mesenchymal stem cells from the third passage (P3) to P5 were used for transplantation. Mouse BMSCs (P6) were bought from Cyagen BioTech, and P7 and P8 cells were used for coculture assays.

The 661w cell line was a gift from Dr Muayyad R. Al‐Ubaidi (University of Oklahoma Health Sciences Center, USA). As a mouse photoreceptor‐derived cell line, 661w was commonly used as an in vitro model for photoreceptor damage, because these cells express multiple cell markers of cone photoreceptors.^20^, ^21^ The cells were cultured as described previously in high‐glucose DMEM (HyClone) supplemented with 10% FBS (Gibco) in a humidified atmosphere of 95% air and 5% CO_2_ at 37°C.^22^ These cells were generally passaged by trypsinization at a ratio of 1:6 every 3‐4 days. For experiments performed in hypoxic conditions, culture dishes and plates were cultured in a sealed, anaerobic workstation (Ruskin Technologies), where the hypoxic condition (1% O_2_, 94% N_2_ and 5% CO_2_), temperature (37°C) and humidity (90%) were kept constant.^23^, ^24^ To inhibit autophagy, 3‐MA (Sigma) was added to the culture medium to achieve a final concentration of 10 mmol/L 1 hour before the experiments as previously suggested.^25^

### 2.2 In vitro coculture experiments

A Transwell coculture system was used to explore the protective effects of mouse BMSCs under the hypoxic condition. 661w cells were seeded and grown in 24‐well (2 × 10^5^ cells/well) or 6‐well (1 × 10^6^ cells/well) Transwell plates (Corning). Bone marrow mesenchymal stem cells at the third passaged were seeded onto the upper or lower well of the Transwell system and cultured for 2 days. The 661w cells were randomly divided into the following groups: (a) normal group: no treatment; (b) hypoxia group: 661w cells cultured under the hypoxic condition; (c) hypoxia +3‐MA group: the autophagy inhibitor 3‐MA was added 1 hour before the hypoxia treatment; (d) coculture group: BMSCs cocultured with hypoxic 661w cells; and (e) autophagy‐inhibited (inhibited) group: the autophagy inhibitor 3‐MA was added 1 hour before the coculturing began under hypoxia.

### 2.3 Cell viability assay

The MTS assay (CellTiter 96^®^ AQueous One Solution, Promega) was performed to determine the viability of cells in each group. Briefly, CellTiter 96^®^ AQueous One Solution was added (20 μL/well), and the cells were incubated for 1 hour at 37°C in a 5% (v/v) CO_2_ atmosphere. The optical density at 490 nm (OD_490_) of each well was measured with a multifunctional microplate reader (VarioSkan, Thermo), using a background control as the blank. Each group was assayed in triplicate, and this test was performed for three times. The cell viability was expressed as the percentage of the control.

### 2.4 Mitochondrial transmembrane potential (ΔΨm)

JC‐1 (Sigma) staining was used to determine **Δ**Ψm. Briefly, 661w cells in each group were incubated with a JC‐1 working solution (0.3 μg/mL) at 37°C in the dark for 30 minutes and observed under a confocal microscope (Olympus FV1000).

### 2.5 Cell apoptosis and necrosis assay

Apoptosis was detected with an Alexa Fluor^®^ 488 annexin V/Dead Cell Apoptosis Kit (Invitrogen) according to the manufacturer's instructions. Briefly, the cells were harvested, washed twice with PBS (PBS, 0.01 mol/L, pH 7.4) and resuspended in annexin‐binding buffer. Then, the Alexa Fluor^®^ 488 Annexin V and PI working solution were added to each 100 μL of sample. After incubation for 15 minutes at room temperature in the dark, 400 μL of 1× annexin‐binding buffer was added, and the samples were kept on ice before the apoptotic rates were analysed by flow cytometry (Beckman Coulter EPICS XL‐MCL).

### 2.6 Caspase activity assays

Caspase‐3 activity was measured in 12‐well plates by the luminescent method using the Caspase‐Glo Assay kit (Keygene Biotech). Cells of each group were washed twice with PBS, before 1 mL/well of fresh growth medium and 1 μL of detection solution were added. After incubation for 30 minutes in the dark, luminescence was measured by the microplate reader VarioSkan.

### 2.7 Monodansylcadaverine staining

Monodansylcadaverine (MDC; Sigma) staining was used to detect autophagosomes in cells of each group. After washing with PBS for three times, cells were incubated with MDC (0.05 mmol/L in PBS) at 37°C for 60 minutes, patterns of green fluorescence were detected by confocal microscopy at an excitation wavelength of 492 nm and an emission wavelength of 520 nm.

### 2.8 Animals

All animal experiments were performed in accordance with the Association for Research in Vision and Ophthalmology (ARVO) Statement for the Use of Animals in Ophthalmic and Vision Research and approved by the Ethics Committee of the Second Hospital of Jilin University in Changchun, China (No. 2017‐068). The female Wistar rats (200‐250 g) used in this study were purchased from the Experimental Animal Center of Jilin University (Changchun, China). Changes in the retina were observed at 4 time points after transplantation: 3 days, 1 week, 2 weeks and 4 weeks (when the retinas were reattached).

### 2.9 Retinal detachment induction and treatment administration

RD in rats was induced as previously described.^2^ Briefly, an anterior chamber puncture was performed via the corneal limbus to lower the intraocular pressure, and approximately one half of the retina was detached mechanically by the subretinal injection (1 mm posterior to limbus) of 1% sodium hyaluronate (Alcon) into the subretinal space. All animals with surgical complications were excluded.

One group (n = 5) of rats received a subretinal transplantation of BMSCs into the detached site (BMSC group), and another group (n = 5) received a PBS injection, which served as the cell vehicle (NC group). The model rats receiving no treatment served as the blank group. The anaesthetized rats were treated with topical anaesthetic drops, and their pupils were then dilated. Under a microscope (Topcon), a 30‐gauge needle was used to create a self‐sealing scleral tunnel after the anterior chamber puncture. A 33‐gauge needle connected to a 10 μL Hamilton syringe (Hamilton) was inserted into the subretinal space through the sclerotomy, and 1 × 10^5^ cells (3.0 μL total volume) were injected.

### 2.10 Tissue preparation

Five rats in each group were euthanized with an overdose of sodium pentobarbital at various time points. An 8‐0 vicryl suture (Johnson & Johnson) was attached to the nasal conjunctiva of each eye to mark the anatomical position. Eyes were enucleated and fixed at 4°C overnight with 4% paraformaldehyde (Sigma) in PBS. The fixed eyes were embedded in Optimal Cutting Temperature (OCT) media (Sakura Finetec) and cut into 8‐μm sagittal sections through the optic nerve head on a cryostat (Leica) frozen at −25°C.

### 2.11 TUNEL assay and ONL thickness ratio evaluation

Prior to staining with fluorescent dye DAPI (Sigma), which was often used to show the nuclei of cells, a terminal dUTP nick‐end labelling (TUNEL) assay (In Situ Cell Death Detection Kit; Roche Diagnostics) was performed according to the manufacturer's instructions. The centre of the detached retina was determined and photographed. The number of TUNEL‐positive cells in the photoreceptor layer was counted at 12 points in nine sections per eye. Cells in the outer nuclear layer (ONL) costained by both TUNEL and DAPI were considered positive, and the number of TUNEL‐positive cells compared to the total ONL area was deemed the apoptosis ratio. The relative thickness of the ONL was defined as (ONL thickness/total thickness in the detached retina)/(ONL thickness/total thickness in the attached retina) in the central area of the detached retina, as well as the attached retina, and was measured by Image‐Pro Plus software version 6.0 (MediaCybernetics Inc).

### 2.12 Immunohistochemistry

Three sections were incubated with 1% BSA for 1 hour to block non‐specific binding. Subsequently, the sections were incubated with primary antibody (the concentrations are listed in Table 1) overnight at 4°C. Alexa Fluor 633‐conjugated goat anti‐mouse IgG or Alexa Fluor 488‐conjugated goat anti‐mouse IgG (1:800, Invitrogen) was used as the secondary antibody, and the mixture was incubated at room temperature for 1 hour. The nucleus was counterstained with DAPI, and images of the retina were taken with an epifluorescence microscope (Olympus IX71).

**Table 1:** Dilutions and manufacturers of antibodies used in this study

Primary antibodies	Concentration	Manufacturers
Rhodopsin	1:1000	Abcam, Cambridge, UK
Caspase‐8	1:500	CST, Massachusetts, USA
Caspase‐9	1:400	Abcam, Cambridge, UK
β‐Actin	1:5000	CMCTAG, Wisconsin, USA
LC3B	1:400	Novus Biologicals, Colorado, USA
p62	1:1000	Abcam, Cambridge, UK

### 2.13 Western blot analysis

Western blotting was performed to evaluate the key players in the apoptotic cascade and photoreceptor and glial cell markers to delineate the protective mechanisms of the cells. Retinas from eyes in those three groups were dissected from the RPE choroid at 3 days, 1 week, 2 weeks and 4 weeks after BMSC transplantation. Proteins were collected according to a method described previously.^8^ The concentration of each sample was determined using the BCA method. Protein samples were then separated with 10% SDS‐PAGE gels and transferred to PVDF membranes (0.45‐μm pores; Millipore). After blocking with 5% non‐fat milk in TBST, the membranes were incubated with a primary antibody (Table 1) at 4°C overnight. The membranes were then incubated for 60 minutes at room temperature with an HRP‐labelled anti‐rabbit or anti‐mouse secondary antibody (1:5000, Millipore). Protein bands were detected by enhanced chemiluminescence (ECL, Millipore) and exposure to film (Aermei‐film). Densitometry analysis was performed with Image‐Pro Plus, and protein levels were normalized to those of actin.

### 2.14 Statistics

All statistical analyses were performed using SPSS 19.0 software (IBM, IL). Numerical data are presented as the means and standard deviation (±SD). Differences between two groups were analysed using *t* tests or Mann‐Whitney *U* tests, while multiple groups were analysed by one‐way ANOVA or Kruskal‐Wallis tests. *P* < .05 was considered a significant difference.

## 3 RESULTS

### 3.1 Autophagy plays a protective role in hypoxia‐treated 661w cells

**Figure 1:**
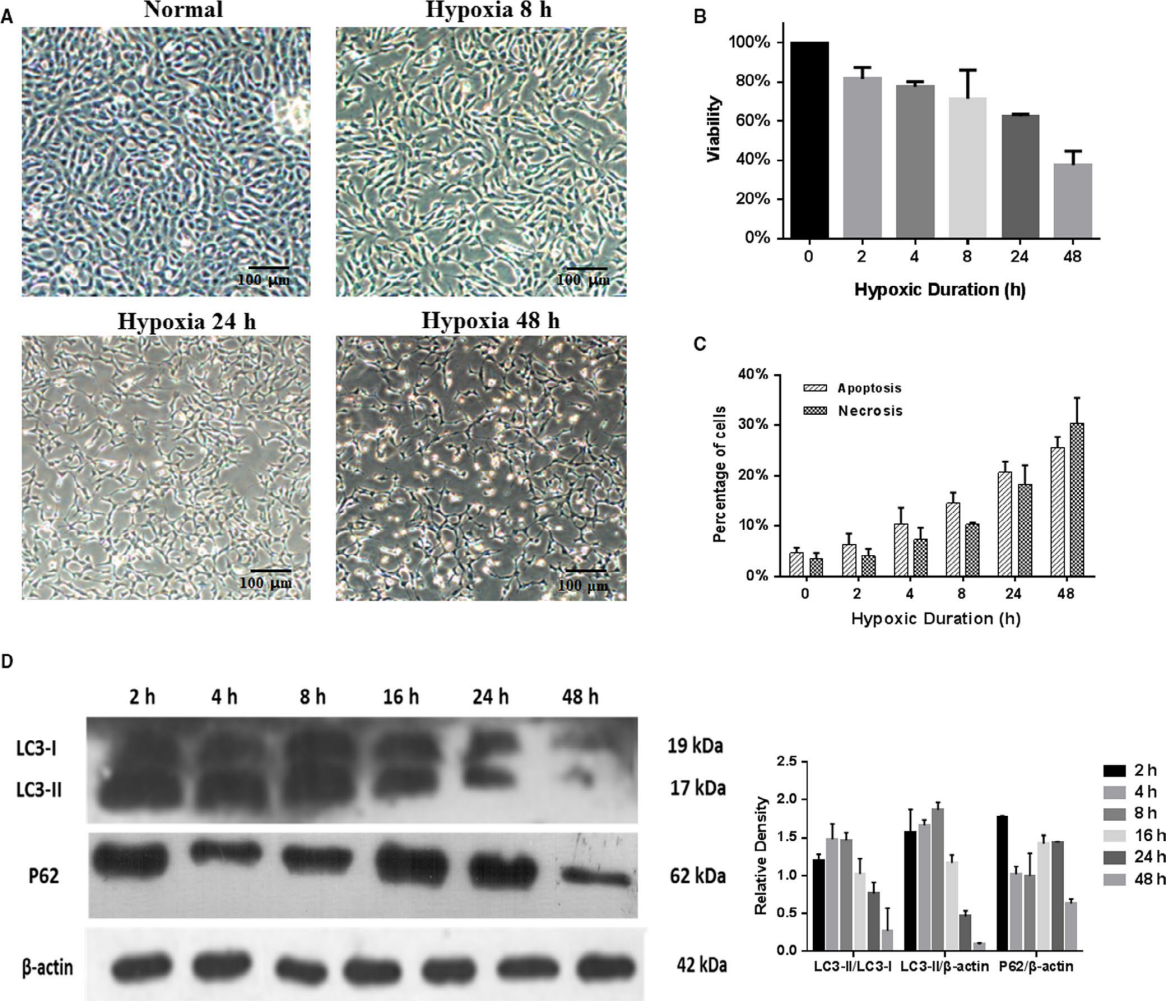
Hypoxia changes morphology, viability and apoptosis of 661w cells. (A) 661w cells began to show morphological changes after being cultured under hypoxic conditions for 8 h, and the changes worsened after 24 h and 48 h, with some cells becoming rounded and floating. Magnification: 4×. (B) The cell viability decreased as the hypoxic time extended, dropping to less than 50% of that of normal cells after 48 h. (C) Apoptosis and necrosis in 661w cells under hypoxia. The percentage of apoptotic cells, as well as that of necrotic cells, was mildly increased after 2 h in hypoxia and gradually increased as the low‐oxygen exposure extended; at 48 h, the proportion of necrotic cells surpassed that of apoptotic cells, indicating necrosis herein became mainly responsible for the decreased cell viability. (D) The expression of LC3‐I, LC3‐II and p62 in 661w cells exposed to hypoxia for 2, 4, 8, 16, 24 and 48 h, by Western blot. Autophagy increased in the first 8 h, and then, autophagy decreased. These assays were repeated for three times

When cultured under hypoxic conditions, 661w cells showed significant morphological changes, especially after 24 hours, and some cells were even rounded and floating (Figure 1A). The cell viability decreased as the hypoxic time extended, falling below 50% of that of normal cells after 48 hours (Figure 1B). The rate of cell apoptosis mildly increased after 2 hours in hypoxia and gradually increased as the low‐oxygen exposure extended; at 48 hours, the proportion of necrotic cells surpassed that of apoptotic cells, and necrosis became the main reason underlying the observed decrease in viability (Figure 1C).

**Figure 2:**
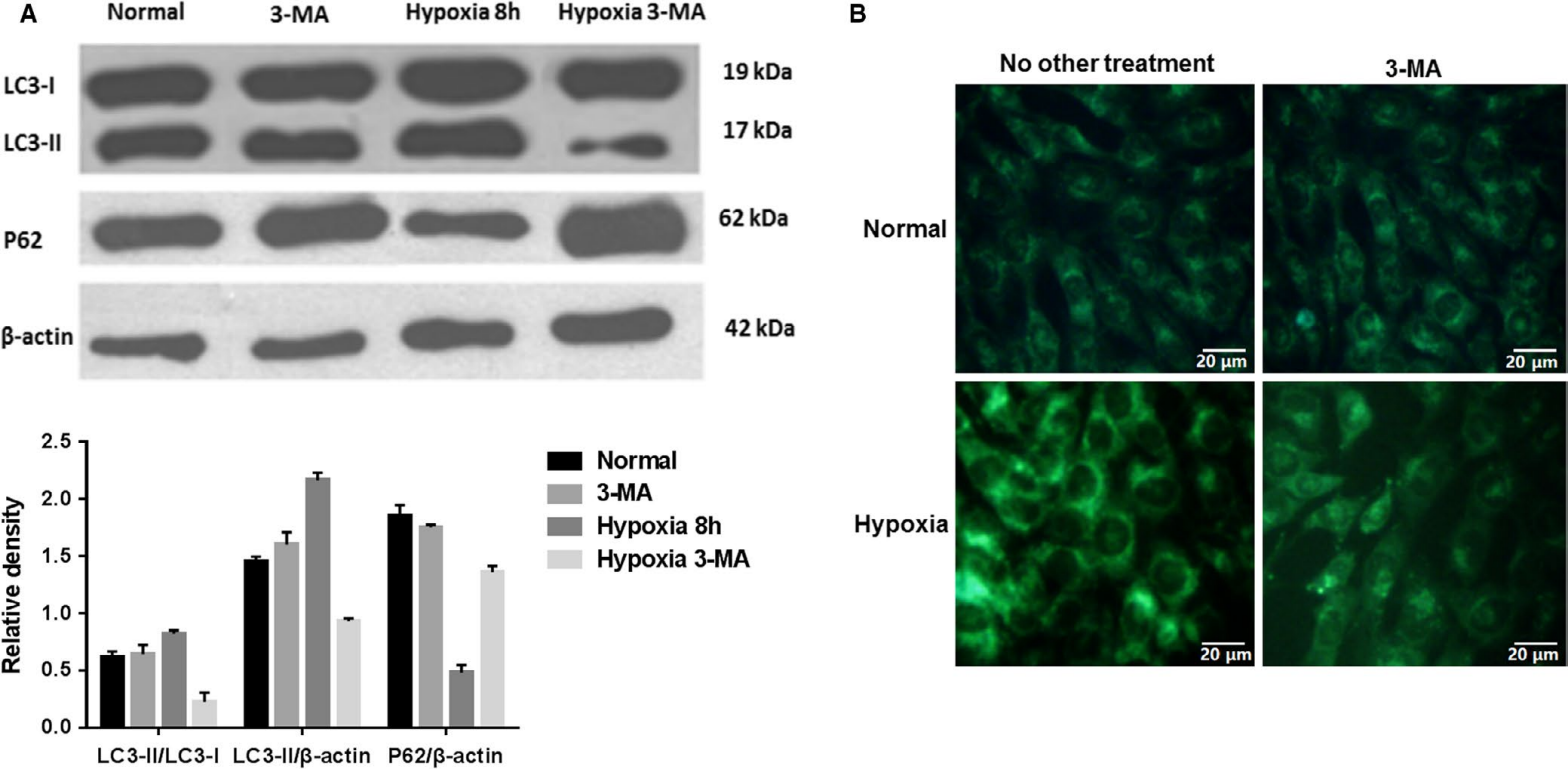
Autophagy was elevated in hypoxic 661w cells. (a) Under normoxic conditions, cells treated with 3‐MA or not did not show significant differences in autophagy. After 8 h in hypoxia, the expression of the autophagy‐related protein LC3‐II was increased, while p62 expression decreased. And this trend could be reversed by the inhibitor 3‐MA. (B) MDC staining (green puncta revealed MDC‐labelled autophagosomes) showed that autophagy was up‐regulated in the hypoxia group and suppressed in hypoxic cells treated with the inhibitor 3‐MA. Magnification: 20×. These assays were repeated for three times

**Figure 3:**
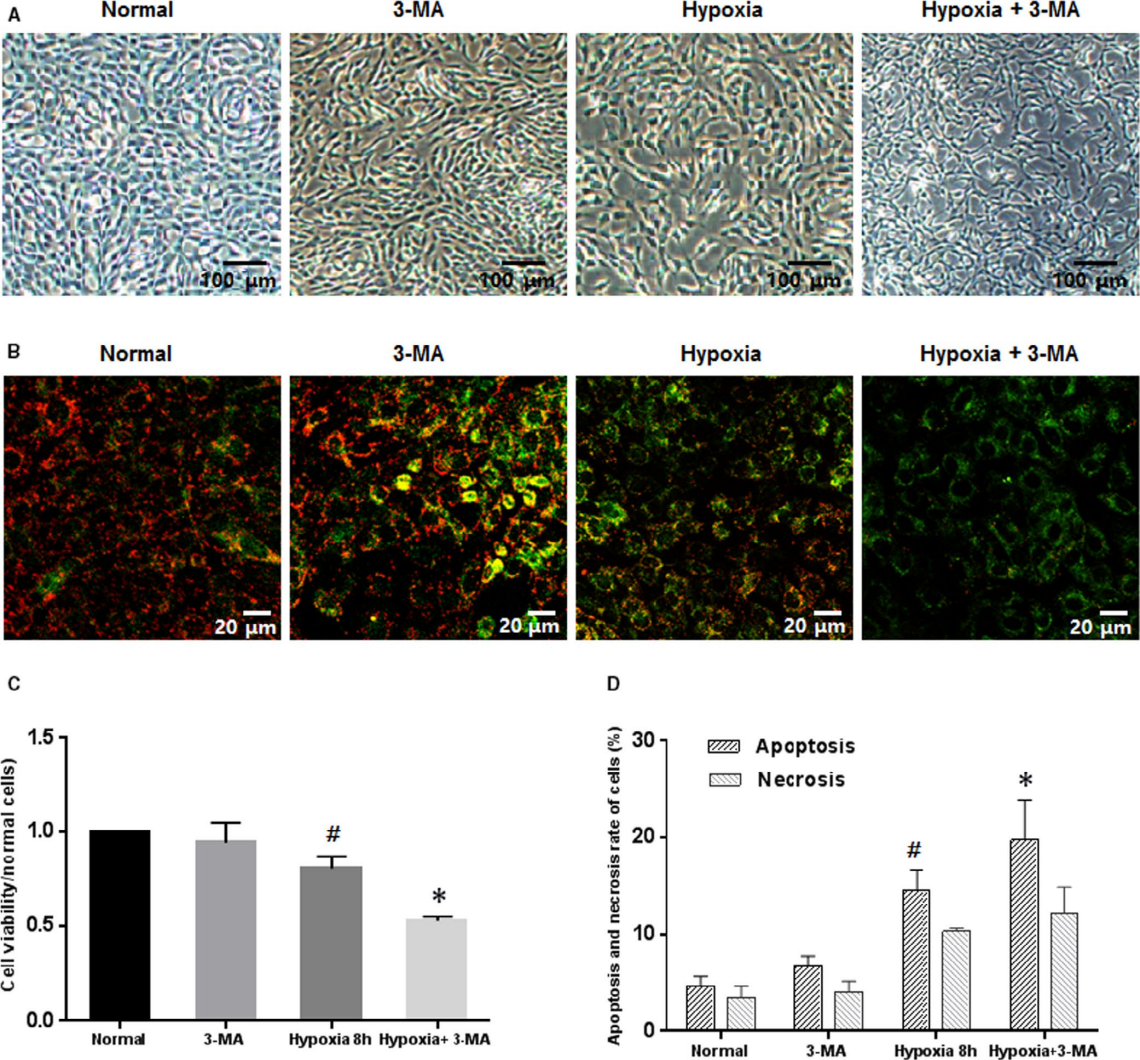
Inhibiting autophagy under hypoxic conditions increased apoptosis and decreased cell viability. (A) The morphologies of 661w cells treated with 3‐MA under hypoxia altered obviously, while those exposed to 3‐MA under normoxic condition did not. Magnification: 4×. (b) **Δ**Ψm analyses by JC‐1 staining. The **Δ**Ψm in cells cultured in hypoxia was diminished, as revealed by increased green fluorescence, and adding 3‐MA into cultures under hypoxic conditions further exacerbate the **Δ**Ψm alteration. Magnification: 20×. (C and D) When autophagy was inhibited in 661w cells under hypoxic conditions, cell viability was decreased while apoptosis was increased. #: *P* < .05, compared to normal and 3‐MA cells, *: *P* < .05, compared to hypoxic cells. These assays were repeated for three times

The hypoxia condition was previously shown to induce autophagy in 661w cells.^24^ We confirmed this in our study (Figure 1D) and further inhibited autophagy with 3‐MA to study its protective role in hypoxic 661w cells. Cells were incubated with 3‐MA, an autophagosome‐lysosome fusion inhibitor, 1 hour before the hypoxic conditions were introduced. When 3‐MA was added to the normoxic culture, no significance difference was observed between the two groups (Figure 2). However, after 8 hours in hypoxia, both autophagy‐related protein expression and MDC staining (green puncta revealed MDC‐labelled autophagosomes) showed that autophagy was up‐regulated in the hypoxia group and suppressed in hypoxic cells treated with the 3‐MA inhibitor (Figure 2). Upon analysing the cellular morphology, viability, apoptosis rate and **Δ**Ψm, hypoxia was shown to exert a detrimental effect on the cells. When autophagy was inhibited, the cells showed no significant changes under the normoxic condition. Compared with those in the hypoxia group, cells in the hypoxia +3‐MA group were more morphologically altered and had a lower viability and a higher apoptotic rate (*P* < .05, Figure 3). This indicated that inhibiting autophagy in a hypoxic environment increases cell apoptosis and decreases cell viability, and autophagy may play a protective role in the early stage of hypoxia stress.

### 3.2 In vitro coculturing with BMSCs showed a positive effect on hypoxic cells partially by increasing autophagy

**Figure 4:**
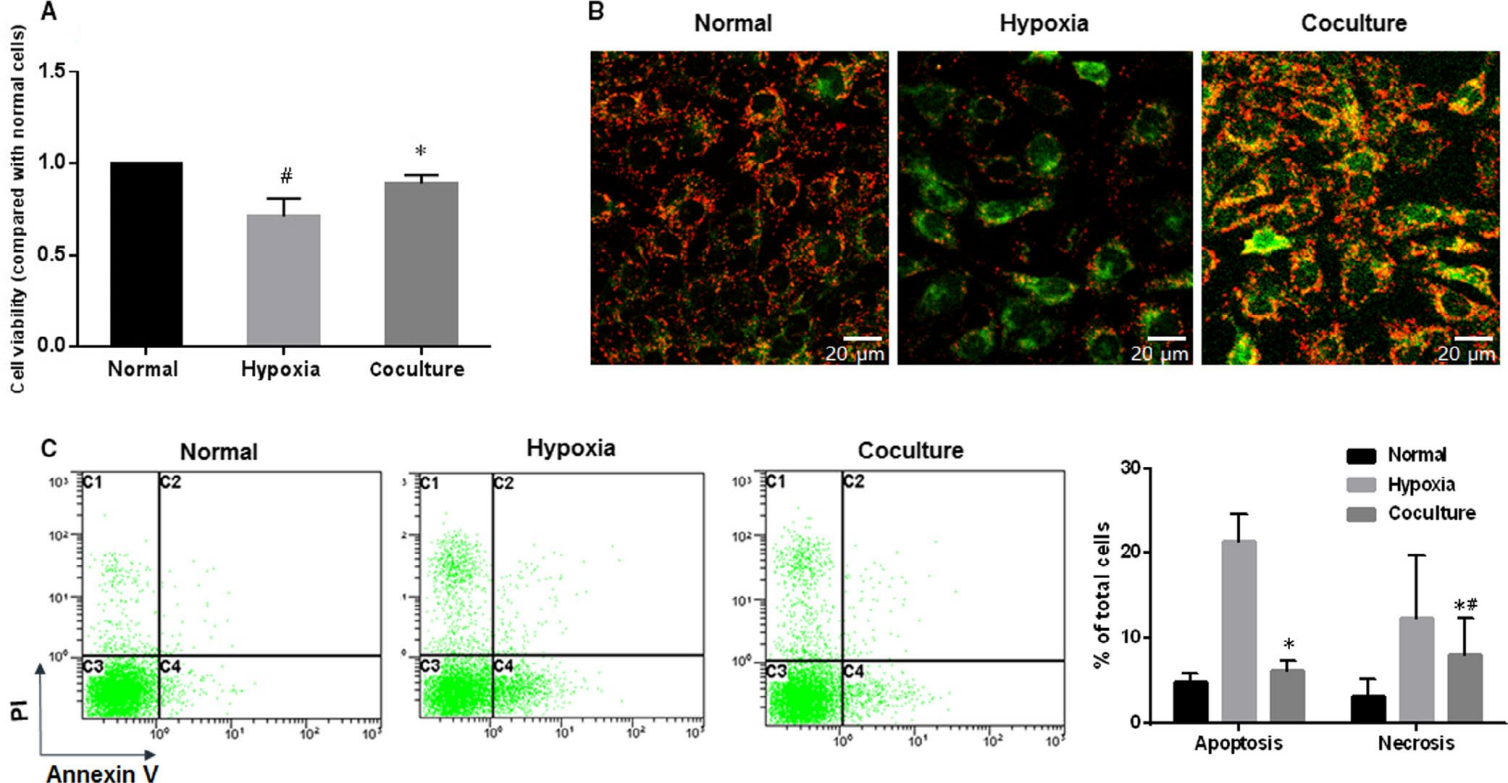
Coculturing with BMSCs promotes better cell morphology and fewer apoptotic cells. (A) Culturing in hypoxic conditions reduced the cell viability of 661w cells, which could be rescued by coculturing with BMSCs, although slightly lower than that of normal cells. (B) Red fluorescence indicated JC‐1 in normal cells with a normal **Δ**Ψm, while green fluorescence indicated a low **Δ**Ψm in hypoxic cells. Magnification: 20×. (C) Coculture with BMSCs decreased the apoptosis and necrosis in 661w cells undergoing hypoxia. These assays were repeated for three times. *: *P* < .05, compared with hypoxic cells. #: *P* < .05, compared with normal cells

After culturing under the hypoxic condition, obvious morphological alterations of 661w cells were found, as they displayed less adhesion and more cell death than cells under the normal condition. However, when the cells were cocultured with BMSCs (coculture group), significantly more cells with normal morphology and increased cell viability (although slightly lower than that of normal cells) were observed (Figure 4A).

As shown in Figure 4B where red fluorescence indicated JC‐1 in normal cells with a high **Δ**Ψm, whereas green fluorescence indicated a low **Δ**Ψm in hypoxic cells, coculture with BMSCs could inhibit the **Δ**Ψm decrease induced by hypoxia treatment. Meanwhile, Annexin V‐488/PI dual staining was applied to detect the effects of BMSCs on the apoptosis and necrosis of 661w cells. Also, both the apoptosis and the necrosis increased by hypoxia could be lowered by coculture with BMSCs (Figure 4C).

**Figure 5:**
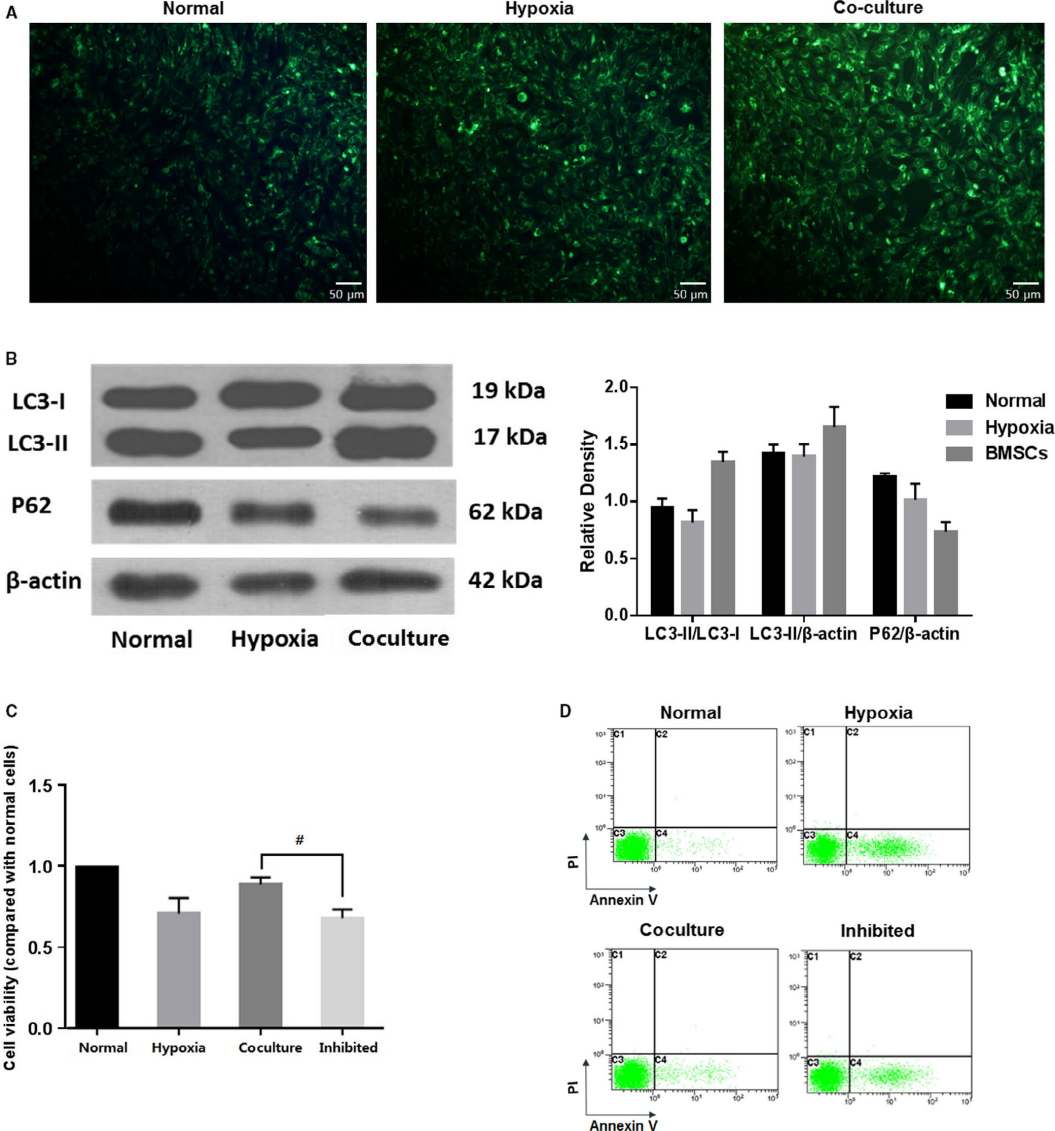
Coculturing with BMSCs increased autophagy in 661w cells, and the effect of BMSCs was weakened when autophagy was inhibited. (A) MDC staining showed higher number of autophagosome in hypoxic cells, as compared to normal cells, and the autophagosome was further increased in the cocultured group. Magnification: 20×. (B) In the cocultured group, the expression of LC3II and the ratio of LC3II/LC3I were obviously increased, while the expression of p62 was decreased, compared with those in the hypoxia and normal groups. (C) 3‐MA, an autophagy inhibitor strongly diminished the protective effects of BMSCs. In the autophagy‐inhibited coculture (inhibited) group, the cell viability was decreased to nearly that in the hypoxia group. These assays were repeated for three times. *: *P* < .05, compared with the other two groups. #: *P* < .05, compared with cocultured cells

Next, we examined the effects of BMSCs on autophagy in 661w cells. Coculturing with BMSCs induced more intense autophagy compared with that in the hypoxia group, as determined by MDC staining (Figure 5A) and Western blot analysis (Figure 5B). The number of autophagosomes increased in the hypoxic cells, and this number was even higher in the cocultured group (Figure 5A). Meanwhile, the level of LC3‐II, as well as the LC3‐II/LC3‐I ratio which indicates the degree of autophagy, was elevated, and the expression of p62 was reduced in the coculture group compared to those in the hypoxia group (Figure 5B). To further investigate the role of autophagy, we treated 661w cells with 3‐MA, an autophagy inhibitor. 3‐MA treatment strongly diminished the protective effects of BMSCs as evidenced by cell viability assessment. In the autophagy‐inhibited coculture (inhibited) group, the viability of cells exposed to 3‐MA was decreased to nearly that in the hypoxia group (Figure 5C). In another word, 3‐MA abrogated the increase in viability conferred by BMSC coculture.

### 3.3 BMSC migrated under retina and the transplantation attenuated photoreceptor damage in retinal detachment

**Figure 6:**
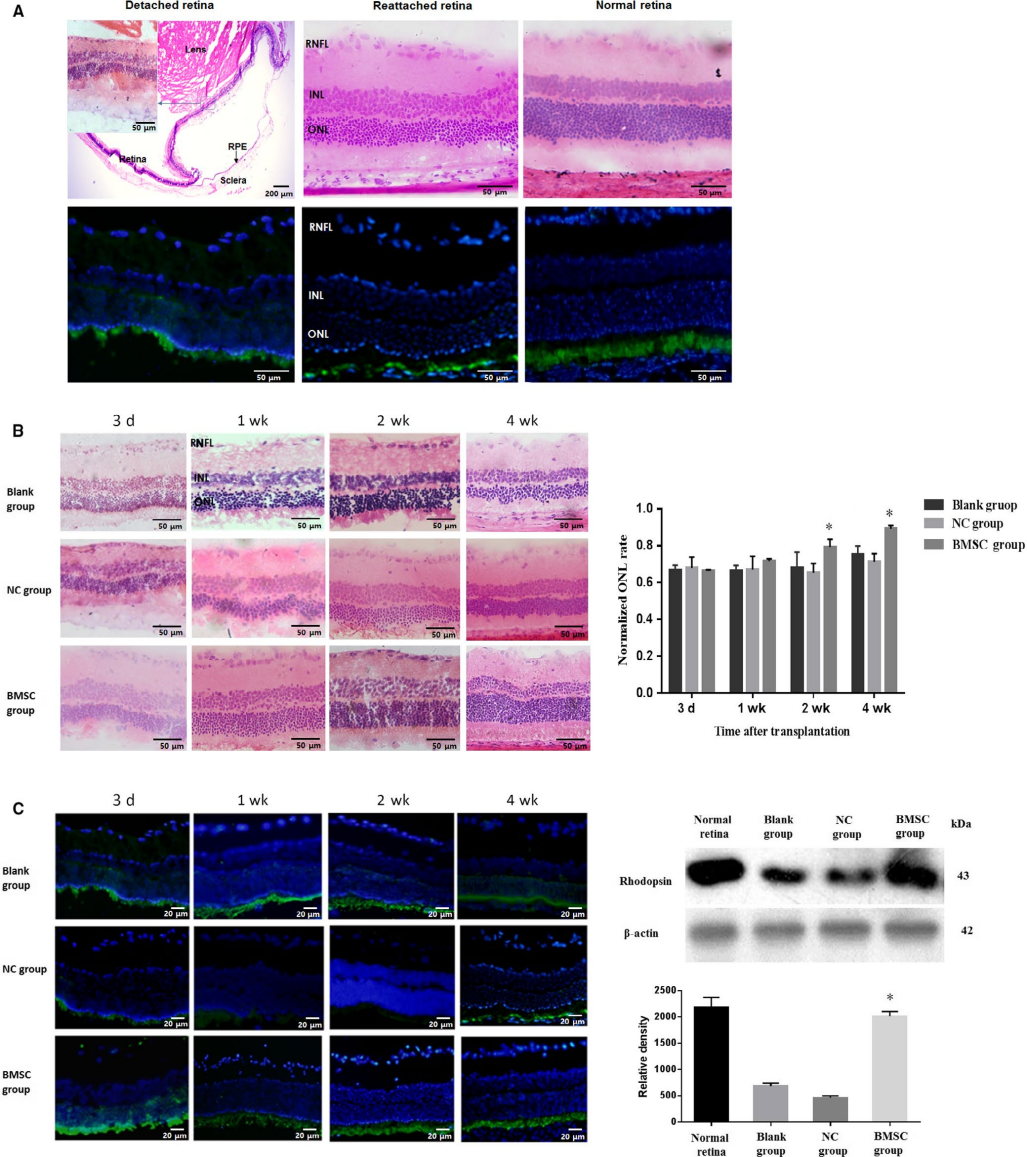
BMSC transplantation ameliorated cell death during retinal detachment. (A) In the detached retina, decreased ONL thickness, shortened photoreceptor outer segments and disordered arrangement were observed after 1 d of RD. Even after the retina was reattached, damage was still observable, as the whole retinal layer was thinned and undulated, especially in the ONL, and scattered nuclei were observed. Compared to normal retina, detached and reattached retinas were disordered and showed decreased expression of rhodopsin, as revealed by weak green fluorescence. Magnification: the photograph of detached retina from HE staining was 4×, while others (including inset) were 20×. (B) Histological changes were ameliorated in the BMSC‐transplanted group, as a thicker ONL was observed, especially at the 2 and 4 wk after transplantation. Magnification: 20×. (C) Photoreceptors were more regularly arranged and thicker in BMSC‐transplanted groups, as detected by immunohistochemistry (magnification: 40×), with rhodopsin expression being increased to nearly normal levels after the retina was reattached. These assays were repeated for three times. *: *P* < .05, compared with the blank and NC groups

RD damaged the retina, as thinning ONL thickness, shortened photoreceptor outer segments, and disordered arrangement was observed after 1 day of RD. Even 28 days after the retina was reattached, the damage was still observable. The entire retinal layer was thinned and undulated, especially the ONL, which had scattered nuclei. When immunofluorescence analysis was applied to detect the expression of rhodopsin, which reflects the photoreceptor state, detached and reattached retinas were disordered and showed weaker fluorescence compared with the normal retina (Figure 6A). However, these histological changes were ameliorated in the BMSC transplantation group, with a thicker ONL being observed, especially at 2 and 4 weeks after transplantation. Furthermore, photoreceptors were more regularly arranged and thicker, as revealed by immunohistochemistry, with rhodopsin expression being increased to nearly a normal level after the retina was reattached (Figure 6B,C). The BMSCs were marked to track their destiny after transplantation in the RD model. After transplantation, these cells could be found under the retina where they can migrate. These results were shown in Figure S2.

### 3.4 BMSCs inhibit retinal detachment‐induced photoreceptor apoptosis

**Figure 7:**
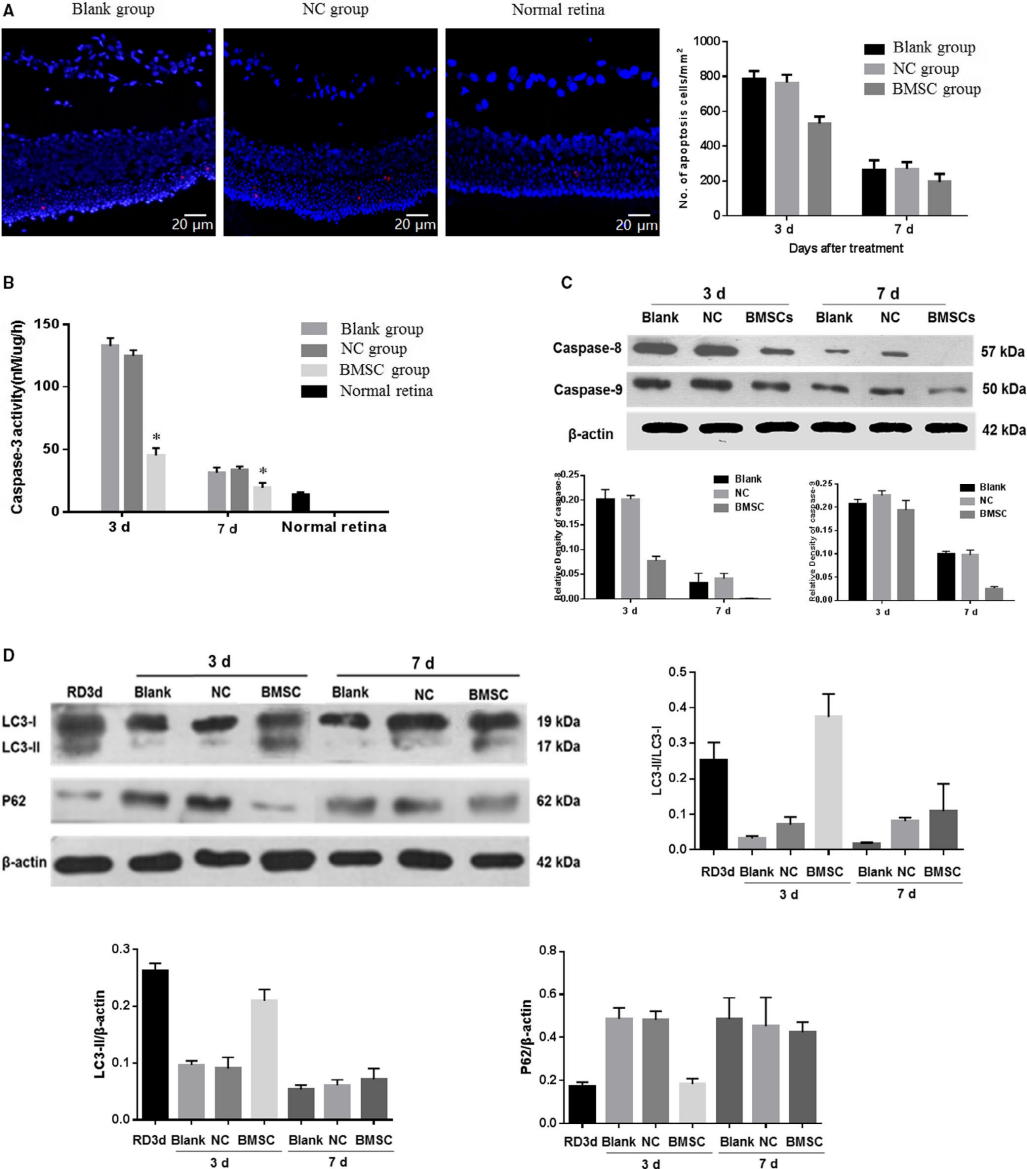
BMSC transplantation attenuated apoptosis and activated autophagy in photoreceptors in retinal detachment. (A) TUNEL staining showed that the subretinal transplantation of BMSCs significantly reduced the number of TUNEL‐positive cells in the ONL at 3 (cells/mm^2^, *P* < .05) and 7 d (cells/mm^2^, *P* < .05) after treatment compared to those in the blank and NC groups. Magnification: 40×. (B and C) At 3 d after transplantation, the treated group showed significantly lower cleaved caspase‐3 levels than the other two groups (*P* < .05), and at 1 wk after transplantation, this level decreased to nearly that in the normal retina. Significant differences in caspase‐8 and cleaved caspase‐9 levels were also found between retinas treated with BMSCs and the other two groups. (D) The expression of the microtubule‐associated protein LC3‐II was decreased, and p62 expression was increased at 6 and 10 d after RD compared with those at 3 d after RD, suggesting that autophagy levels peaks on day 3 after RD. Three days after treatment, significantly increased LC3‐II expression and decreased p62 levels were found in the BMSC‐treated group compared to those in the other two groups. However, after 7 d post‐treatment, the levels did not differ significantly among the groups. These assays were repeated for three times

First, we assessed photoreceptor cell death after RD by TUNEL staining. The subretinal transplantation of BMSCs significantly reduced the number of TUNEL‐positive cells in the ONL on day 3 (cells/mm^2^, *P* < .05) and day 7 (cells/mm^2^, *P* < .05) after treatment compared to those in the blank group. However, after 14 days, the number of apoptotic cells reduced rapidly, and no difference was observable between the groups (Figure 7A).

Caspase family members, which are central regulators of apoptosis, were shown to be activated in the retina in the early stage of RD.^26^ Three days after treatment, the BMSC group showed significantly lower cleaved caspase‐3 levels than the other two groups (*P* < .05), and 1 week after transplantation, this level decreased to nearly that in the normal retina (Figure 7B). Significant differences in caspase‐8 and cleaved caspase‐9 levels were also observed between retinas treated with BMSCs and the other two groups (*P* < .05; Figure 7C). As the activity and protein levels of caspases decreased to nearly the normal level in the later stage, the three groups showed no significant differences.

### 3.5 Autophagy in photoreceptors could be activated by BMSCs

To study the effect of BMSCs on autophagy in RD, the expression levels of autophagic signalling markers were examined by Western blot analysis. The expression of the microtubule‐associated protein LC3‐II was decreased, and p62 expression was increased at 6 and 10 days after RD compared with that at 3 days after RD, suggesting that autophagy peaks 3 days after retinal detachment. Three days after BMSC transplantation, significantly increased LC3‐II expression and decreased levels of p62 were observed in the BMSC‐treated group compared to those in the other two groups (Figure 7D). However, after 7 days post‐RD, the levels of LC3‐II and p62 among the groups were not significantly altered. Together, these results indicated that transplanted BMSCs may protect the retina by promoting autophagy.

## 4 DISCUSSION

In this study, we showed the beneficial effects of BMSCs on retinal photoreceptor damage. The intraocular transplantation of BMSCs ameliorated the apoptosis of photoreceptors and preserved the morphology and structure of detached retinas, partly via activating autophagy to help local cells survive the hypoxia and denutrition in the early stage of RD. In vitro analysis conducted simultaneously showed that coculturing with BMSCs also played a positive role in hypoxia‐induced damage in 661w, a photoreceptor cell line, by inducing the expression of LC3‐II, suggesting that BMSCs exert an autophagy‐inducing effect.

Bone marrow mesenchymal stem cells, a type of adult multipotent and immunoprivileged cell, are a promising cell source of cytotherapy and have been studied in various retinal diseases.^27^, ^28^, ^29^, ^30^ In animal models of retinitis pigmentosa and retina light damage, intravitreal or subretinal transplantation of BMSCs expressing bFGF or BDNF reduced the number of TUNEL‐positive cells in the ONL and prevented photoreceptor cell loss and differentiated forward the RPE cell and photoreceptor; in age‐related macular degeneration, BMSC transplantation protected various retinal layers and expressed RPE cell markers.^29^, ^31^, ^32^ In this study, the retina, especially the photoreceptors, was damaged by RD, and 661w cells simultaneously showed significant morphological and cell viability changes when cultured under hypoxic conditions which partly imitate oxygen‐lacking situation in the retinal detachment.^24^ Retinas reattached with the subretinal transplantation of BMSCs had a thicker ONL, more regularly arranged photoreceptors, and nearly normal expression levels of rhodopsin. Bone marrow mesenchymal stem cells preserved the structure of detached retinas in vivo and hypoxic photoreceptor cells in vitro. The mechanisms underlying the protective effects were also analysed in this study.

Both intrinsic and extrinsic apoptotic pathways are activated in photoreceptor cells in experimental RD and hypoxia.^1^ Photoreceptor could be protected by inhibiting apoptosis (through blocking cytochrome c release and caspase‐3 activation, or by suppressing the Fas‐signalling pathway), including deletion of anti‐apoptotic Bax, use of small peptide Met12, neurotrophins and compounds like edaravone and tauroursodeoxycholic acid.^1^, ^8^, ^33^, ^34^ Intravitreal delivery of BMSCs also attenuated apoptosis by decreasing caspase‐3 in ischaemic rat retina.^17^ Apoptosis was repressed by BMSCs in this study, as coculturing with BMSCs ameliorated morphological changes and apoptotic cells while simultaneously significantly decreased the expression of apoptotic‐related proteins. Meanwhile, the subretinal transplantation of BMSCs significantly reduced the number of TUNEL‐positive cells in the ONL of the detached retina. The anti‐apoptotic effect of BMSCs was also demonstrated in several tissues; in ischaemic myocardial cells, damaged liver cells and neural cells, BMSCs helped to reduce apoptosis and promote matrix remodelling, and the mechanisms underlying this result were thought to be cytokines produced by the paracrine effect of the BMSCs.^12^, ^30^ Differentiation was also found to be another important aspect, as the transplanted BMSCs expressed several functional cell markers; however, the functions of the differentiated cells remain controversial.^35^, ^36^, ^37^ Recently, studies have shown that BMSCs can regulate autophagy.^15^, ^16^

Autophagy, one of the important mechanisms underlying survival and cellular homoeostasis maintenance, functions by decomposing cellular composition under stresses, such as starvation and hypoxia. Autophagy was shown to not only result in cell death but also play a role in cell survival.^38^ Activated autophagy was found in RPE and endothelial cells cultured under high‐glucose stress, and inhibiting autophagy led to the activation of inflammatory responses and cell death.^25^, ^39^ In an RD model, autophagy peaked at 3 days and decreased at 7 days after RPE‐photoreceptor separation was induced, and played a role in regulating the level of photoreceptor apoptosis in a Fas‐dependent way.^40^ When prolonged and augmented by calpain or TNF‐α inhibitor, autophagy could significantly reduce photoreceptor apoptosis and protect retina.^38^, ^41^ In our in vitro study, autophagy in 661w cells was induced under hypoxic conditions, which was consistent with previous researches of others,^24^ and inhibiting autophagy with 3‐MA resulted in more apoptotic cells. Autophagy and apoptosis, both the activation of which are dependent on Fas, are interconnected, and autophagy in photoreceptors in RD could negatively regulate the caspase‐dependent apoptosis, while inhibiting autophagy in retina could increase the activation of caspase‐8.^38^, ^40^, ^42^, ^43^ In our study, the decrease in caspase‐8 in Fas‐mediated extrinsic apoptotic signalling was more obvious than caspase‐9, suggesting that the apoptosis‐attenuating effect of BMSCs may be exerted through inhibiting extrinsic apoptosis.

Autophagy was also found to be the mechanism by which stem cells play a protective role and improve their own reproductive potential. In damaged liver cells, MSC ameliorated liver fibrosis via activating autophagy,^16^ and human placenta‐derived mesenchymal stem cells also promoted hepatic regeneration by up‐regulating HIF‐1α and activating autophagy.^44^ BMSCs also attenuated lung injury by increasing the autophagy level via the PI3K/Akt signalling pathway.^15^ In this study, coculturing with BMSCs induced more intense autophagy than that in the hypoxia group. To further investigate the role of autophagy, 3‐MA treatment, which inhibited autophagy, strongly diminished the protective effects of BMSCs, as determined by assessing cellular morphology, viability and apoptosis. The paracrine mechanism or exosome may involve in the autophagy induction, and this should be further studied. In vivo, autophagy was activated in the BMSC‐treated group 3 days after treatment, but the autophagy level was not significantly changed after 7 days, indicating that BMSCs may promote autophagy in the early stages after transplantation in detached retinas.

## 5 CONCLUSIONS

Injury caused by the hypoxic environment surrounding photoreceptor cells can lead to decreased cell viability, which is accompanied by more apoptotic cells. Autophagy is simultaneously up‐regulated, and inhibiting autophagy results in increased cell death, indicating an important role of autophagy activation in cell survival in the early stages of hypoxic stress. BMSCs, via activating autophagy, exert a protective effect against cell damage and apoptosis. Subretinal BMSC transplantation can significantly reduce photoreceptor cell death and promote morphological restoration in detached retinas, through reducing retinal cell apoptosis and activating autophagy shortly after transplantation to survive the low‐oxygen and malnutrition environment after periods of RD. Further studies are needed to explore the underlying mechanisms and pathways through which autophagy is activated by BMSCs and to elucidate the details necessary for clinical applications.

## CONFLICT OF INTEREST

The authors have no competing interests to declare.

## AUTHOR CONTRIBUTIONS

XL and JX performed the research. XL and LY analysed the data and wrote the paper. YL, YH and ZL contributed to the acquisition and analysis of data. YZ and GS designed the research study and revised the paper.

## Data Availability

The data used to support the findings of this study are available from the corresponding author upon request.
