# Supplementary figures and images for: Bone marrow mesenchymal stem cells enhance autophagy and help protect cells under hypoxic and retinal detachment conditions
Source: J Cell Mol Med. 2020 Jan 30;24(6):3346–58. doi: 10.1111/jcmm.15008 (PMC7131940; doi:10.1111/jcmm.15008)

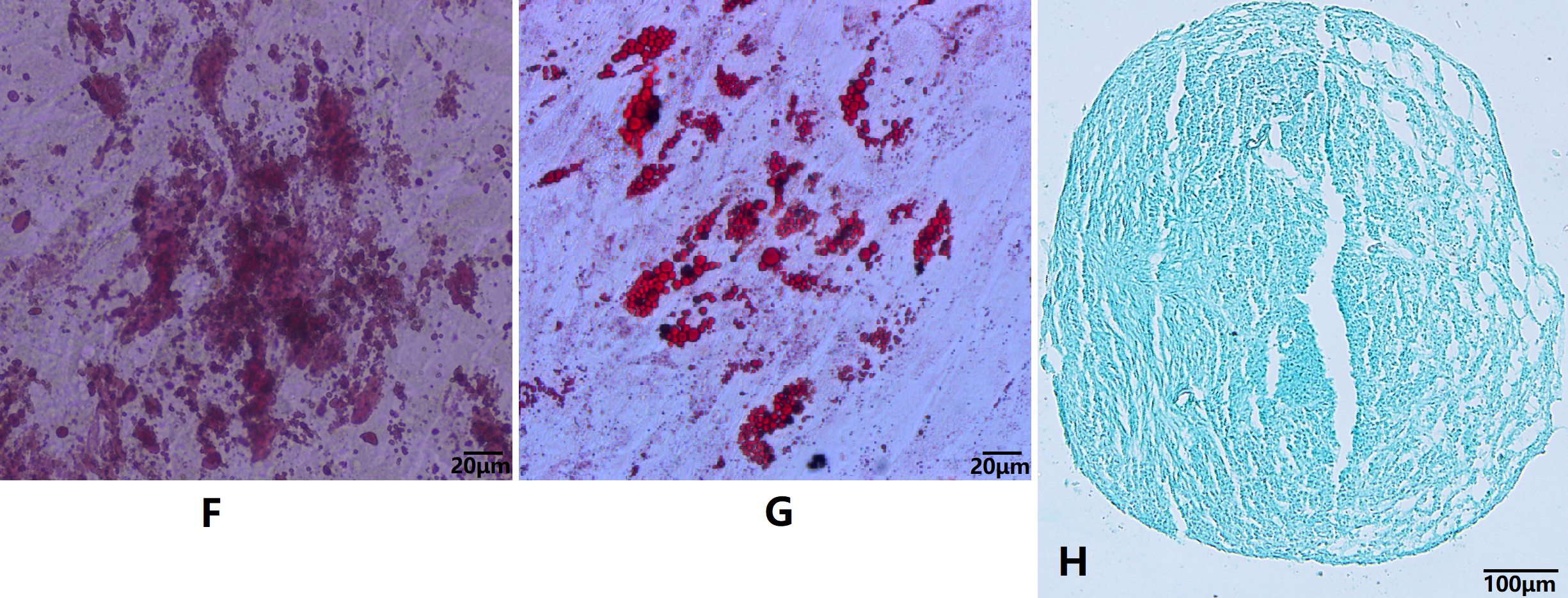

Supplement: Supplementary file 1 [file JCMM-24-3346-s001.tif]

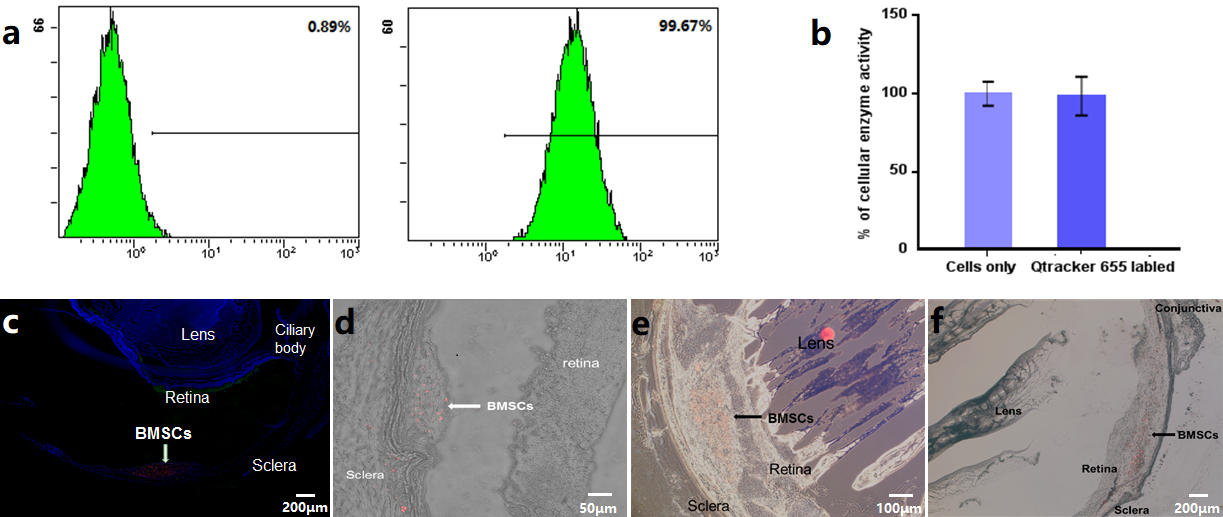

Supplement: Supplementary file 2 [file JCMM-24-3346-s002.tif]
