# Supplementary material for: Bone marrow mesenchymal stem cells enhance autophagy and help protect cells under hypoxic and retinal detachment conditions
Source: J Cell Mol Med. 2020 Jan 30;24(6):3346–58. doi: 10.1111/jcmm.15008 (PMC7131940; doi:10.1111/jcmm.15008)

**Supplementary Materials**

**Supplementary Figure 1: Identification of BMSCs by cell marker analysis using FCM (A-E) and by three-line *in vitro* differentiation (F-H).**

(a) Cells were collected and centrifuged, FITC or Cy3 marked CD 90, CD105, CD34 and CD45 were added and incubated in 37℃ for 1 hour. After washing and resuspending in PBS, they were tested on flow cytometry (Backman, USA). 96.26% of the cells were CD90 positive (B), 97.82% in CD105 (C), while the CD45 positive cells were 0.82% (D)，and CD34 were 1.51% (E). A was negative control. Immunofluorescence assay were also used in identification and the results were not shown.

(b) Osteogenic differentiation (F), chondrogenic differentiation (G) and adipogenesis (H) of isolated cells. Cells at a density of 4x10^4^ cells/mL were seeded into 6-well plates and exposed to inducing medium for different periods. Then, cells were fixed with 4% paraformaldehyde for 20-60 minutes and stained with Alizarin Red S (F, magnification: 20X), Oil Red O (G, 20X), or Alcian Blue (H, 4X) for individual duration before pictures were obtained from the camera coupled with inverted microscope. These assays were repeated for three times.


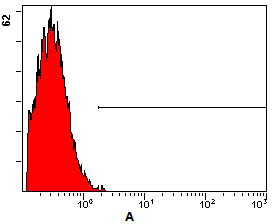

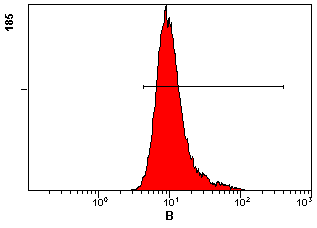

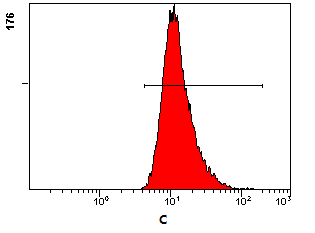

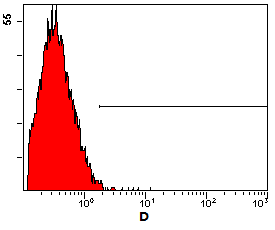

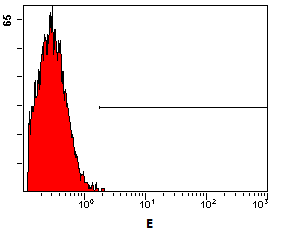


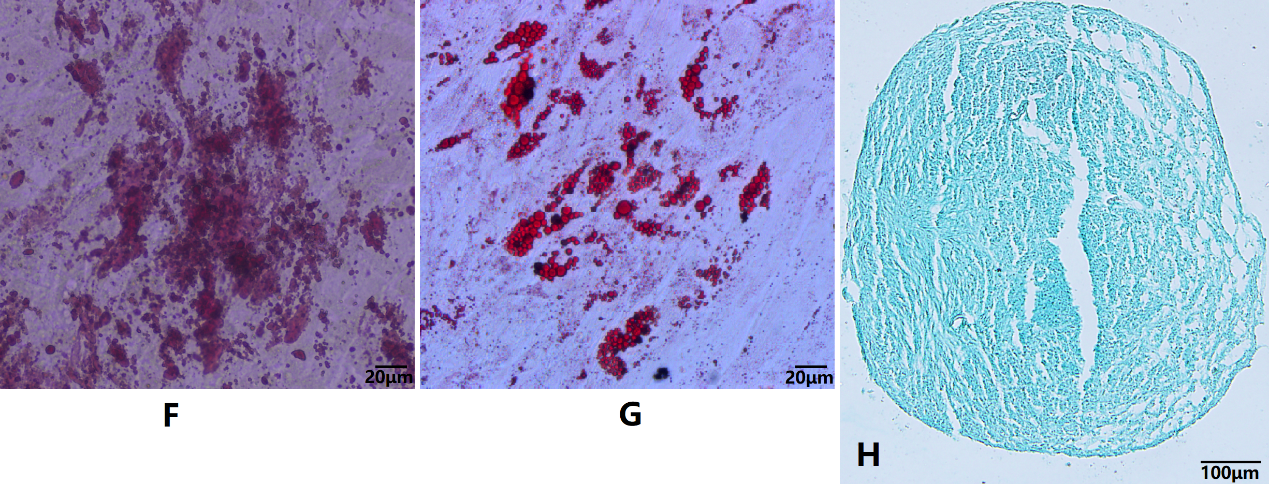


**Supplementary Figure 2: Cell marking and tracking after transplantation.** Before transplantation, the cells were labelled with Qtracker ® 655 (Invitrogen, Canada) following the manufacturer’s instructions and the rate of labelled cell and the viability were tested by flow cytometry (a) and MTS assay which showed a high rate and nearly unchanged cell viability (b). Three days (c, magnification: 4X), 14d (d, 20X), 21d (e, 10X) and 28d after transplantation (f, 4X), eye balls were enucleated, embedded in OCT media and sagittally sectioned with a thickness of 8 μm. The cryosection were observed and photographed in epifluorescence microscope. The cells could be found in sub-retinal space 3 days after transplantation as a rise mound (the red dots as the arrows pointed, c), and kept still under retina after 28 days (f), which showed no sign of migration to other parts of retina.


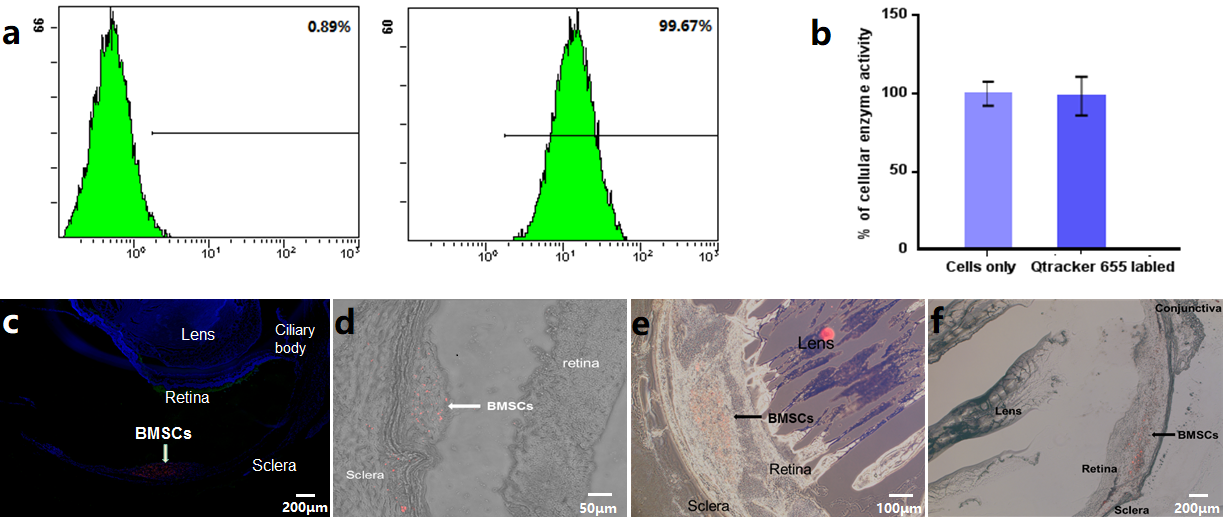

Supplement: Supplementary file 3 [file JCMM-24-3346-s003.docx]
